# Deep infiltrating endometriosis: cine magnetic resonance imaging in the evaluation of uterine contractility

**DOI:** 10.1590/0100-3984.2022.0069

**Published:** 2023

**Authors:** Deborah Monteiro Soares, Leonardo Kayat Bittencourt, Flavia Paiva Proença Lobo Lopes, Marco Aurelio Pinho de Oliveira

**Affiliations:** 1 Radiology Department, Clínica de Diagnóstico por Imagem (CDPI)/DASA, Rio de Janeiro, RJ, Brazil; 2 Radiology Department, University Hospitals and Case Western Reserve University, Cleveland, OH, USA; 3 Radiology Department, Universidade Federal do Rio de Janeiro (UFRJ), Rio de Janeiro, RJ, Brazil; 4 Department of Gynecology, Universidade do Estado do Rio de Janeiro (UERJ), Rio de Janeiro, RJ, Brazil

**Keywords:** Uterus/physiology, Uterine contraction, Infertility, Magnetic resonance imaging, cine, Endometriosis, Adenomyosis, Útero/fisiologia, Contração uterina, Infertilidade, Cine-ressonância magnética, Endometriose, Adenomiose

## Abstract

**Objective:**

To evaluate uterine function by using cine magnetic resonance imaging to visualize the contractile movements of the uterus in patients with and without deep infiltrating endometriosis (with or without associated adenomyosis).

**Materials and Methods:**

This was a prospective case-control study. The study sample comprised 43 women: 18 in the case group and 25 in the control group. We performed cine magnetic resonance imaging in a 3.0 T scanner, focusing on the presence, direction, and frequency of uterine peristalsis.

**Results:**

The frequency of uterine peristalsis was higher in the case group than in the control group, in the periovulatory phase (3.83 vs. 2.44 peristaltic waves in two minutes) and luteal phase (1.20 vs. 0.91 peristaltic waves in two minutes). However, those differences were not statistically significant. There was a significant difference between the patients with adenomyosis and those without in terms of the frequency of peristalsis during the late follicular/periovulatory phase (0.8 vs. 3.18 peristaltic waves in two minutes; *p* < 0.05).

**Conclusion:**

The frequency of uterine peristalsis appears to be higher during the periovulatory and luteal phases in patients with deep infiltrating endometriosis, whereas it appears to be significantly lower during the late follicular/periovulatory phase in patients with adenomyosis. Both of those effects could have a negative impact on sperm transport and on the early stages of fertilization.

## Abstract (pt)

**Objetivo:**

Avaliar a função uterina mediante visualização dos movimentos contráteis do útero por meio de cine-ressonância magnética em pacientes com e sem endometriose infiltrativa profunda (com ou sem adenomiose associada).

**Materiais e Métodos:**

Estudo caso-controle prospectivo. A amostra foi composta por 43 mulheres, sendo 18 mulheres no grupo caso e 25 mulheres no grupo controle. A cine-ressonância magnética foi realizada com magneto 3.0 T, com foco na presença, direção e frequência do peristaltismo uterino.

**Resultados:**

O peristaltismo uterino foi mais frequente nas pacientes do grupo endometriose do que no grupo controle na fase periovulatória (3,83 × 2,44 peristalses em dois minutos) e lútea (1,20 × 0,91 peristalse em dois minutos). No entanto, esses resultados não foram estatisticamente significantes. Nas pacientes com adenomiose, observou-se redução significativa na frequência de peristaltismo durante a primeira fase do ciclo menstrual (3,18 × 0,8 peristalses; *p* < 0,05).

**Conclusão:**

A frequência de peristaltismo uterino parece estar aumentada durante a fase periovulatória e lútea em pacientes com endometriose infiltrativa profunda e significativamente reduzida em pacientes com adenomiose durante a primeira fase do ciclo menstrual. Ambos os efeitos têm potencial de interferir negativamente no transporte de espermatozoides e nos primeiros estágios de fecundação.

## INTRODUCTION

Endometriosis is a common gynecological pathology, characterized by the presence of stroma, endometrial epithelium, or both, outside the uterus^(1)^. It can affect several sites and is known as deep infiltrating endometriosis (DIE) when there is infiltration of the wall of the pelvic organ^(2)^.

Endometriosis is the most common gynecological pathology identified among women undergoing laparoscopic examination for the investigation of infertility^(3)^. Regarding infertility, some factors related to the uterus have been studied, including uterine peristalsis, which plays a vital role in female fertility. The uterus undergoes rhythmic contractions, which help transport sperm to the fallopian tubes and support the maintenance of early pregnancy^(4-7)^. Adenomyosis can also contribute to infertility because it affects sperm transport by altering the muscle fiber architecture in the uterus, thus impairing endometrial function and local receptivity^(8-10)^.

Magnetic resonance imaging (MRI) is one of the best noninvasive methods for the diagnosis of DIE and adenomyosis^(11,12)^. For the diagnosis of DIE, MRI in a 3.0-T scanner has a sensitivity of 96.3%, a specificity of 100%, and a negative predictive value of 100%^(11)^. Through the use of cine MRI, it is also possible to assess contractile movements by visualizing uterine peristalsis^(6,7,13)^.

The aim of this study was to evaluate uterine peristalsis and its characteristics, using cine MRI in 3.0 T scanners, comparing patients with and without DIE, as well as patients with and without adenomyosis. Our hypothesis was that uterine peristalsis would be altered in patients with DIE, perhaps especially in those with adenomyosis, and that cine MRI would be able to identify patients with altered uterine peristalsis, as well as to inform strategies for increasing fertility.

## MATERIALS AND METHODS

This was a prospective case-control study, carried out between May 2018 and March 2019 in the Gynecology Department of the Pedro Ernesto University Hospital, operated by the Universidade do Estado do Rio de Janeiro in the city of Rio de Janeiro, Brazil, in partnership with the Diagnostic Imaging Clinic of Diagnósticos da América, also in the city of Rio de Janeiro. The study was approved by the Research Ethics Committee of the Universidade do Estado do Rio de Janeiro (Reference no. 2.513.972), and all participating patients gave written informed consent.

The inclusion criteria were being ≥ 18 years of age, being in menacme, and being scheduled to undergo MRI of the pelvis. The initial group of volunteers comprised 64 patients between 18 and 45 years of age. Patients who had had a hysterectomy were excluded, as were those who were pregnant, those who were using a hormonal contraceptive or intrauterine device, those who had amenorrhea or were in the menstrual phase, and those for whom MRI was contraindicated. The volunteers underwent MRI examination in a 3.0-T scanner, with a standard protocol for assessing the pelvis and an additional cine MRI sequence. Patients in whom the MRI scan was of insufficient quality would also be excluded. On the basis of the MRI findings, the patients were divided into two groups: those with DIE (case group); and those without (control group).

In the literature, DIE is defined as the presence of implants or masses that appear on MRI as hypointense areas or hyperintense foci on T1- or T2-weighted images at multiple locations in the pelvis^(14,15)^. For the diagnosis of DIE through MRI, we used the criteria established by Bazot et al.^(16)^.

Of the 64 patients initially included, 28 were in the case group and 36 were in the control group. A total of 21 patients were excluded: nine (five from the case group and four from the control group) because they had amenorrhea; three (two patients from the case group and one from the control group) because they were using (oral) hormonal contraceptives; four (two from each group) because they were in the menstrual phase; and five (one from the case group and four from the control group) because they could not remember the date of the last menstruation. Therefore, the final sample comprised 43 patients: 18 in the case group and 25 in the control group. In the case group, MRI revealed DIE affecting the following sites (two or more sites were affected in 10 cases): torus uterinus (n = 6); uterosacral ligaments (n = 8); vagina (n = 2); rectovaginal septum (n = 2); rectosigmoid (n = 7); pouch of Douglas (n = 3); parametrium (n = 1); bladder (n = 6); and round ligaments (n = 3). Ovarian endometriomas were identified in seven cases.

Of the 43 patients evaluated, 15 were in the periovulatory phase, 22 were in the luteal phase, and six were in the initial follicular phase. We defined the initial follicular phase as the period from day 1 to day 10 of the menstrual cycle, the periovulatory phase as the period from day 10 to day 18, and the luteal phase as the period from day 18 to day 28 (the standard menstrual cycle was considered to be 28 days for all patients).

### MRI protocol

All MRI examinations were performed in a 3.0-T scanner (Prisma; Siemens Medical Systems, Erlangen, Germany). All patients were also submitted to the cine MRI protocol. With the patients breathing normally, a total of 60 serial images of the mid-sagittal plane of the uterus were obtained in half-Fourier acquisition single-shot turbo spin-echo sequences (echo time: 80 ms; field of view: 300 mm; slice thickness: 5 mm; matrix: 512 × 384; and flip angle: 150°), one image being acquired every two seconds over a two-minute period. After those images had been acquired, the patients also underwent MRI of the pelvis with a standard protocol for evaluating endometriosis. Prior to undergoing cine MRI, none of the patients received antispasmodic drugs, because such drugs can interfere with uterine peristalsis.

### Analysis of images acquired by cine MRI

The images acquired by cine MRI were analyzed on an OsiriX digital imaging and communications in medicine-picture archiving and communication system workstation (https://www.osiriximaging.com). The sequences were evaluated by two radiologists with eight and 13 years of experience in the area of gynecology (radiologist A and radiologist B, respectively), working independently, who were blinded to the day of the cycle of the patient imaged. In cases of disagreement, the review and final evaluation were carried out by radiologist A, who had greater expertise in the analysis of cine MR sequences to assess contractions.

The following variables were measured in both groups: the presence or absence of peristalsis; the frequency of peristaltic waves per two-minute interval; the direction of the peristaltic waves (cervico-fundal or fundo-cervical); and the presence or absence of sustained uterine contractions. In both groups, adenomyosis was identified by radiologist A, on the basis of the pelvic MRI findings, according to the criteria established in the literature^(17)^: junctional zone ≥ 12 mm; maximal junctional zone thickness/myometrial thickness ratio > 40%; a regular, asymmetrical increase in uterine volume; or hyperintense signal foci on T1- or T2-weighted images of the myometrium; and no leiomyomas. For the evaluation of uterine contractility, cine MRI sequences were evaluated visually, in a dynamic mode, at 12× faster than real time.

The uterus has an inherent contractility, visible on imaging as two distinct patterns of myometrial contraction, which vary throughout the menstrual cycle. One pattern, known as sustained contraction, involves the entire myometrium, whereas the other, known as uterine peristalsis, occurs only in the innermost myometrium^(6)^.

**Figure 1:**
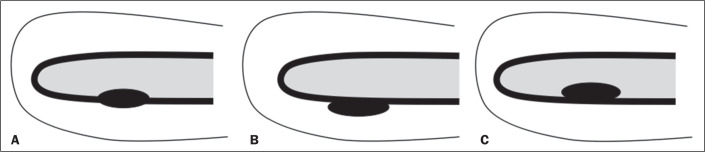
Recognition of peristalsis on cine MRI^(10)^. A,B: Low signal intensity wave conduction on the longitudinal axis within the junctional zone. C: Movements of depression of the endometrium.

**Figure 2:**
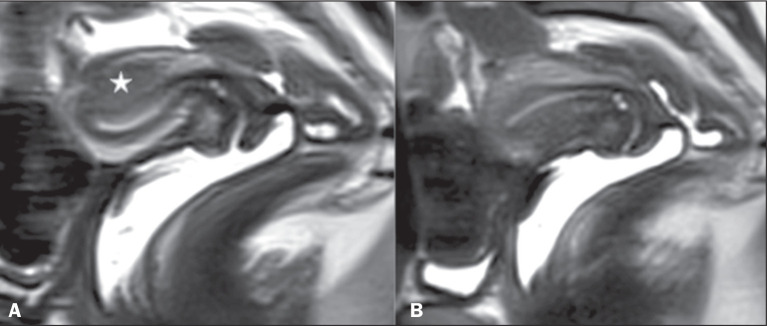
Sustained contraction: T2-weighted images in the sagittal plane. A: Poorly defined focal area with low signal in the myometrium (star) during the dynamic phase (cine mode), which disappear later in the static sequence (B).

The presence of peristalsis (Figure 1) was defined on the basis of the findings described in previous studies^(6,7)^. Wave conduction, when perceptible, was characterized as cervico-fundal or fundo-cervical. The total number of waves within two minutes was recorded. As depicted in Figure 2, sustained uterine contractions were defined as areas of low signal intensity on a T2-weighted sequence, sustained throughout the cine acquisition^(6,7)^.

### Statistical analysis

Categorical variables are reported as absolute and relative frequencies, whereas continuous variables are reported as mean and standard error of the mean. Intergroup comparisons were analyzed by independent t-tests for continuous variables and by chi-square tests for categorical variables. Two-tailed *p*-values less than 0.05 were considered statistically significant. The level of agreement between the two radiologists was assessed by intraclass correlation coefficient for the continuous variables and by Cohen’s kappa coefficient for the categorical variables.

## RESULTS

**Figure 3:**
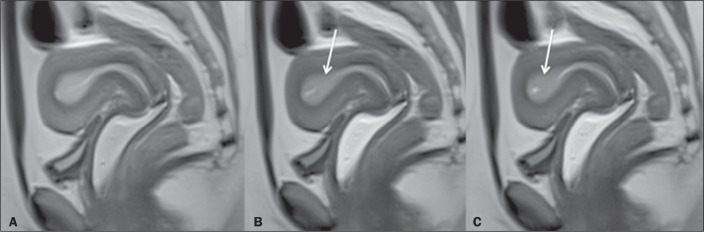
A 35-year-old woman (control group). Cine MRI showing uterine peristalsis during the periovulatory phase. A: Uterus at rest between peristalses. B,C: The waves were rhythmic and conspicuous, in the cervico-fundal direction (arrows).

As shown in Table 1, there were no statistically significant differences between the two groups in terms of the mean age, body mass index, parity, or infertility. In the periovulatory phase (Figure 3), peristaltic contractions were more common and more frequent among the patients in the case group than among those in the control group, although the difference was not statistically significant. In the luteal phase, the frequency of peristalsis was also higher among the case group patients. For the frequency of peristalsis, the intraclass correlation coefficient was 0.94 (95% CI: 0.91-0.96), which indicates excellent agreement. For the detection of uterine peristaltic activity, Cohen’s kappa coefficient was 0.75 (95% CI: 0.87-0.99), which indicates good agreement.

**Table 1:** Characteristics of the study groups.

Characteristic	Cases (n = 18)	Controls (n = 25)	*P*
Age (years), mean ± SEM	36.6 ± 6.0	34.6 ± 6.5	0.31
Parity (≥ two children), n (%)	2 (11.1)	6 (24.0)	0.26
Infertility, n (%)	6 (33.3)	9 (36.0)	1.00
Weight status, n (%) Underweight	2 (11.1)	1 (4.0)	
Normal weight	5 (27.8)	9 (36.0)	0.85
Overweight	8 (44.4)	9 (36.0)	
Obesity	3 (16.7)	6 (24.0)	

SEM, standard error of the mean.

The results of the evaluation of peristalsis in the three phases of the menstrual cycle are summarized in Tables 2 and 3. Peristaltic activity was more common in the periovulatory phase than in the follicular and luteal phases, being observed (all in the cervico-fundal direction) in 12 (80.0%) of the 15 patients who were in the periovulatory phase, compared with two (33.3%) of the six who were in the follicular phase and nine (40.9%) of the 22 who were in the luteal phase (*p* = 0.019). As can be seen in Table 2, peristaltic waves were detected in five (83.3%) of the six case group patients who were in the periovulatory phase, compared with seven (77.8%) of the nine control group patients who were in that same phase (*p* = 1.00). As shown in Table 3, the mean number of peristaltic waves over a two-minute period was higher in the case group than in the control group, in the periovulatory phase (3.83 ± 0.48 vs. 2.44 ± 0.4; *p* = 0.23) and in the luteal phase (1.20 ± 0.56 vs. 0.91 ± 0.2; *p* = 0.73). There was no significant difference between the case and control groups in terms of the direction of the peristaltic waves.

**Table 2:** Presence or absence of peristalsis by cycle phase.

Cycle phase	Peristalsis	Cases (n = 18) n (%)	Controls (n = 25) n (%)	*P*
Follicular	Present	1 (5.5)	1 (4.0)	1.00
	Absent	1 (5.5)	3 (12.0)	
Periovulatory	Present	5 (27.7)	7 (28.0)	1.00
	Absent	1 (5.5)	2 (8.0)	
Luteal	Present	3 (16.6)	6 (24.0)	0.41
	Absent	7 (38.8)	6 (24.0)	

**Table 3:** Frequency of peristaltic waves over a two-minute period.

Cycle phase	Cases (n = 18) Mean ± SEM	Controls (n = 25) Mean ± SEM	*P*
Follicular	1.00 ± 0.33	2.00 ± 0.8	0.67
Periovulatory	3.83 ± 0.48	2.44 ± 0.4	0.23
Luteal	1.20 ± 0.56	0.91 ± 0.2	0.73

SEM, standard error of the mean.

**Figure 4:**
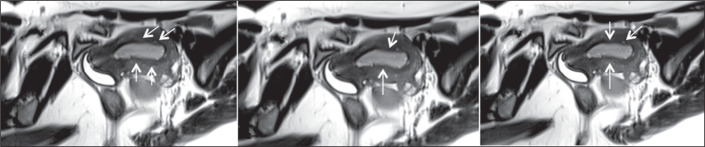
A 38-year-old woman (case group) with DIE, adenomyosis, and infertility (18 months). Cine MRI during the luteal phase, showing frequent, dysrhythmic waves in the cervico-fundal direction (arrows).

**Figure 5:**
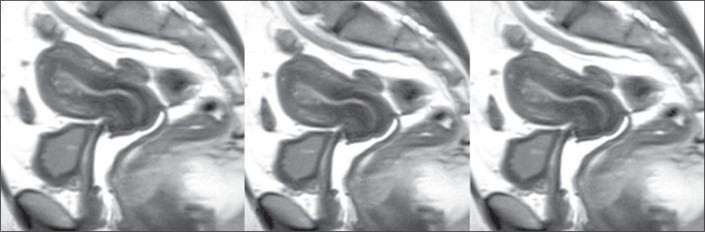
A 39-year-old woman (control group) with adenomyosis. Cine MRI during the follicular phase, showing no uterine peristalsis.

Of the 43 patients in the study sample, 11 (25.5%) had adenomyosis: six (33.3%) of the 18 in the case group and five (20.0%) of the 25 in the control group (*p* = 0.52). In general, peristalsis was less common among the patients with adenomyosis than among those without (Figures 4 and 5). Although the difference not statistically significant, it is noteworthy that, among the patients who were in the periovulatory phase, peristalsis was observed in only one of the four patients with adenomyosis, whereas it was observed in all 11 of the patients without. In addition, the mean number of peristaltic waves over a two-minute period during the late follicular and periovulatory phases was significantly lower among the patients with adenomyosis than among those without: 0.8 vs. 3.18 (*p* = 0.04). Sustained uterine contraction was uncommon, being present in only one case group patient and one control group patient.

Among the patients who were excluded from the analysis, peristalsis was detected in only one of those excluded for having amenorrhea (a control group patient), one of those excluded for using oral contraceptives (a case group patient), and one of those excluded for not remembering the date of the last menstruation (a control group patient). Peristalsis was not detected in any of the patients who were excluded for being in the menstrual phase.

## DISCUSSION

Uterine peristalsis represents an inherent contractility and plays a crucial role in the transport of sperm and in the maintenance of early pregnancy^(6,7,18)^. It is known that peristaltic activity is altered in infertile women with endometriosis. It has been suggested that dysfunction of the physiological mechanism of retrograde uterine peristaltic activity is implicated in the development of endometriosis^(6,10,18,19)^, as well as that changes in peristaltic activity and endometriosis both contribute to infertility. To increase the pregnancy rate in infertility treatments, some studies have used therapy with agents that reduce uterine activity in the luteal phase of induced cycles and have shown a statistically significant difference compared with placebo^(18,20,21)^. To our knowledge, there have been no previous studies using cine MRI in 3.0-T scanners to evaluate uterine peristalsis in patients with and without DIE.

In the present study, we observed that uterine peristalsis in the periovulatory phase was more common in the patients with DIE than in those without. The peristaltic frequency in the periovulatory and luteal phases was also higher in the case group than in the control group. Although not statistically significant, these findings underscore the fact that endometriosis has a negative impact on uterine functional dynamics, triggering potential impairment of fertilization processes. Increased uterine activity during the periovulatory phase, a period of great importance for the reproductive system, has significant potential to impede the transport of sperm for fertilization and, later, to induce expulsion of the embryo. In the luteal phase, that increased contractility could impair embryo implantation or even contribute to the involution of an initial pregnancy, because, during that phase, the uterus needs to be at rest for embryonic development. These results, although not statistically significant, are similar to those in the literature^(19)^.

On the basis of transvaginal ultrasound findings, Leyendecker et al.^(19)^ reported that peristalsis was more common in patients with endometriosis than in controls, in all three phases of the menstrual cycle. The authors concluded that the movements of uterine hyperperistalsis identified in the patients with endometriosis revealed dysfunction within the reproduction process that can contribute to the development of infertility. They also highlighted the increased frequency of peristalsis as the main mechanical cause of infertility, given that it prevents the transport of sperm in the pre-ovulatory period and reduces fertility^(19)^. However, in a study of uterine peristalsis evaluated by 1.5-T cine MRI, Kido et al.^(7)^ found that peristalsis was significantly suppressed during the periovulatory phase in patients with ovarian endometrioma. That result is contrary to our findings of hyperperistalsis and to those of others^(10,19)^, possibly because Kido et al.^(7)^ studied only patients with ovarian endometriomas. Another possibility is that their sample included a greater number of patients with sustained uterine contraction, which has been shown to inhibit uterine peristaltic activity. It should also be noted that those authors evaluated uterine movements in a 1.5-T MRI scanner, which obtains images of slightly lower resolution than those obtained in 3.0-T scanners, which are capable of revealing movements that are more subtle.

In the present study, cervico-fundal peristalsis was more common than was fundo-cervical peristalsis, in both groups, and was the predominant direction during the periovulatory phase. These findings are statistically significant and are consistent with the physiological variation of peristalsis throughout the cycle. In the physiological cycle, the direction of peristalsis is retrograde (cervico-fundal) in the periovulatory phase and anterograde (fundo-cervical) during menstruation^(2,4,22,23)^.

Another important result of our study was that the frequency of peristalsis was significantly lower in the patients with adenomyosis during the first phase of the cycle (day 5 to day 18), which includes the late follicular and periovulatory phases. As previously stated, the mean number of peristaltic waves over a two-minute period was 3.18 in the control group patients, whereas it was only 0.8 in the patients with adenomyosis. That difference was statistically significant and corroborates the findings of other authors, who have shown that adenomyosis reduces uterine contractile activity, which in turn impairs the transport of sperm in the periovulatory period, making it a potential cause of infertility^(14,15)^. In our study sample, sustained uterine contraction was uncommon, possibly because we excluded patients who were in the menstrual phase, in which such contraction is more common.

Our study has some limitations. It was not possible to perform a detailed assessment of the relationships among endometriosis, uterine peristalsis, and fertility. That was due to the small sample size and the limited data on fertility. In addition, we did not obtain images of the same patients in different phases of the menstrual cycle, which could have allowed us to characterize the contractile behavior on an individual basis. There is a need for prospective studies investigating that behavior in patients undergoing infertility treatment. Furthermore, although our results suggest that DIE and adenomyosis have an effect on peristalsis, the small number of cases (only 11 cases of adenomyosis among only 18 cases of DIE) increased the likelihood of a type II error, which would limit the generalizability of the results. Studies involving larger patient samples are needed in order to corroborate our findings. Moreover, there was no surgical confirmation of DIE in our case group patients and it could not definitely be determined that our control group patients were free of endometriosis. Nevertheless, all of the patients included in the study were diagnosed with DIE on the basis of MRI scans evaluated by an experienced radiologist.

## CONCLUSION

Through the use of cine MRI, we were able to demonstrate that the frequency of uterine peristalsis in patients with DIE was higher during the periovulatory and luteal phases of the menstrual cycle, which are crucial periods for sperm transport and embryo implantation. Uterine peristalsis in those phases has great potential to reduce fertility. We also demonstrated that adenomyosis has a significant impact on uterine contractility, being associated with a significant reduction in the frequency of peristalsis in the first phase of the menstrual cycle, thus also impairing the initial fertilization processes. We believe that uterine contractility continues to be a promising target in the treatment of infertility. Its dynamics can be assessed safely, quickly, and reliably using cine MRI in a 3.0-T scanner.

## References

[1] Olive DL, Pritts EA (2001). Treatment of endometriosis. N Engl J Med.

[2] Viganò P, Parazzini F, Somigliana E (2004). Endometriosis: epidemiology and aetiological
factors. Best Pract Res Clin Obstet Gynaecol.

[3] Giudice LC, Swiersz LM, Burney RO, Jameson JL, De Groot LJ (2010). Endocrinology.

[4] Ashrafi M, Sadatmahalleh SJ, Akhoond MR (2016). Evaluation of risk factors associated with endometriosis in
infertile women. Int J Fertil Steril.

[5] Xiao L, Zhang Q, Huang X (2019). Endometrial stromal cell miR-29c-3p regulates uterine
contraction. Reproduction.

[6] Soares DM, Werner Junior H, Bittencourt LK (2019). The role of cine MR imaging in the assessment of uterine
function. Arch Gynecol Obstet.

[7] Kido A, Togashi K, Nishino M (2007). Cine MR imaging of uterine peristalsis in patients with
endometriosis. Eur Radiol.

[8] Barbanti C, Centini G, Lazzeri L (2021). Adenomyosis and infertility: the role of the junctional
zone. Gynecol Endocrinol.

[9] Kunz G, Beil D, Huppert P (2005). Adenomyosis in endometriosis-prevalence and impact on fertility.
Evidence from magnetic resonance imaging. Hum Reprod.

[10] Leyendecker G, Kunz G, Herbertz M (2004). Uterine peristaltic activity and the development of
endometriosis. Ann N Y Acad Sci.

[11] Hottat N, Larrousse C, Anaf V (2009). Endometriosis: contribution of 3.0-T pelvic MR imaging in
preoperative assessment-initial results. Radiology.

[12] Takeuchi M, Matsuzaki K (2011). Adenomyosis: usual and unusual imaging manifestations, pitfalls,
and problem-solving MR imaging techniques. Radiographics.

[13] Bazot M, Bharwani N, Huchon C (2017). European society of urogenital radiology (ESUR) guidelines: MR
imaging of pelvic endometriosis. Eur Radiol.

[14] Campo S, Campo V, Benagiano G (2012). Adenomyosis and infertility. Reprod Biomed Online.

[15] Kissler S, Hamscho N, Zangos S (2006). Uterotubal transport disorder in adenomyosis and endometriosis-a
cause for infertility. BJOG.

[16] Bazot M, Daraï E (2017). Diagnosis of deep endometriosis: clinical examination,
ultrasonography, magnetic resonance imaging, and other
techniques. Fertil Steril.

[17] Bazot M, Daraï E (2018). Role of transvaginal sonography and magnetic resonance imaging in
the diagnosis of uterine adenomyosis. Fertil Steril.

[18] Kuijsters NPM, Methorst WG, Kortenhorst MSQ (2017). Uterine peristalsis and fertility: current knowledge and future
perspectives: a review and meta-analysis. Reprod Biomed Online.

[19] Leyendecker G, Kunz G, Wildt L (1996). Uterine hyperperistalsis and dysperistalsis as dysfunctions of
the mechanism of rapid sperm transport in patients with endometriosis and
infertility. Hum Reprod.

[20] Bellver J, Simón C (2018). Implantation failure of endometrial origin: what is
new?. Curr Opin Obstet Gynecol.

[21] Li J, Chen Y, Wang A (2017). A meta-analysis of atosiban supplementation among patients
undergoing assisted reproduction. Arch Gynecol Obstet.

[22] Bulletti C, De Ziegler D, Rossi S (1997). Abnormal uterine contractility in nonpregnant
women. Ann N Y Acad Sci.

[23] Fanchin R, Righini C, Olivennes F (1998). Uterine contractions at the time of embryo transfer alter
pregnancy rates after in-vitro fertilization. Hum Reprod.

